# Anti-Anaphylactic and Anti-Inflammatory Activities of a Bioactive Alkaloid from the Root Bark of *Plumeria acutifolia* Poir

**Published:** 2011

**Authors:** Vijay alakshmi, Ravi chandiran, Malarkodi Velraj, Hemalatha, Jayakumari

**Affiliations:** *Department of Pharmacognosy, School of Pharmaceutical Sciences, Vels University, Pallavaram, Chennai-117, Tamil Nadu, India.*

**Keywords:** *Plumeria acutifolia*, Plumerianine, Anti-anaphylactic, Anti-inflammatory, Bioactive alkaloid

## Abstract

In the present study, anti-anaphylactic, anti-inflammatory and membrane stabilizing properties of a lupine alkaloid plumerianine (compound 1), isolated from the root bark of *Plumeria acutifolia *Poir were investigated in animal models. The anti-anaphylactic activity of compound 1 (10, 25 and 50 mg/Kg) was studied by using models such as passive cutaneous anaphylaxis, passive paw anaphylaxis and also investigated for its anti-inflammatory activity against the carrageenin induced paw edema and cotton pellet granuloma in albino rats. A dose-dependent beneficial effect was observed on the leakage of Evans Blue dye in skin challenged with antigen and on paw anaphylaxis induced by antiserum. The compound 1 also exhibited a significant (p < 0.01) inhibition of rat paw edema and granuloma tissue formation, including significant protection of RBC against the hemolytic effect of hypotonic solution, an indication of membrane-stabilizing activity. Anti-anaphylactic activity of compound 1 may be possibly due to the inhibition of releasing various inflammatory mediators. Anti-inflammatory activity of compound 1 may be related to the inhibition of the early phase and late phase of inflammatory events.

## Introduction

Allergic disorders are in rise every year and are stated as an endemic disease of the 21^st ^century. Some of the allergic disorders, which may be caused by an allergen originating from immune system, environment, and genes, are asthma, eczema, hay fever, anaphylaxis and autoimmune disorders (1). A number of plants are described in Ayurveda to be used in the treatment of allergic disorders (namely psoriasis, eczema and bronchial asthma), amongst which just a few have not been studied earlier for their antiallergic activities. Studying some of these is the purpose of the present study. On activation, mast cells released immediately the preformed and the *de novo *synthesized mediators such as histamine, proteases, leukotrienes, prostaglandins, and cytokines (2). As a consequence, the acute reactions such as vasodilation, increased vascular permeability and bronchoconstriction were induced. In addition, allergic responses also trigger the influx and activation of a variety of inflammatory cells including eosinophils and lymphocytes. Rapidly released mediators and numerous cytokines produced by mast cells are strongly believed to induce and sustain these responses, which may contribute to chronic inflammation. Inflammation is a normal protective response to tissue injury caused by physical trauma, noxious chemicals or microbiological agents. Inflammation is body’s response to inactivate or destroy the invading organisms, remove irritants and set the stage for tissue repair (3, 4). Inflammation is triggered by the release of chemical mediators from the injured tissues and migrating cells. The specific chemical mediators vary with the type of inflammatory process and include amines (such as histamine and serotonin), lipids (such as prostaglandins) and small peptides (such as kinins) (5).

**Figure 1:**
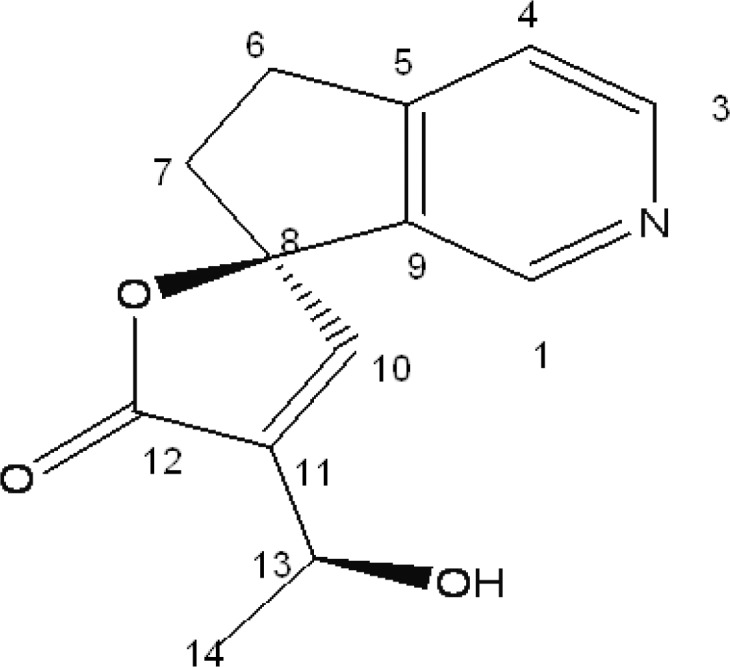
Plumerianine

Conventional anti-inflammatory drugs such as steroidal anti-inflammatory drugs (SAID) and non-steroidal anti-inflammatory drugs (NSAID) are used in healing most of the acute and chronic pain and inflammatory disorders including rheumatoid arthritis. However, long-term use of these agents may produce serious adverse effects. Thus, it is worth developing new plant-derived anti-inflammatory agents with fewer adverse effects. *Plumeria acutifolia *is one of such plants which are reputed to have numerous applications in traditional medicine. The plant has been mentioned in ancient literature as anti-inflammatory, anti-allergic, diuretic, carminative, laxative, anti-ulcer, and useful in treating leprosy and ascites, also possess cytotoxic and anti-microbial activity (6). Earlier phytochemical studies on the root bark had shown the presence of iridoids, tannins and alkaloids (7, 8). In continuation of our studies on medicinal plants for their chemical constituents and biological activities, we isolated plumerianine (structure shown in Figure 1) from the root bark of *Plumeria acutifolia *Poir. In the present paper, we reported the anti-anaphylactic, anti-inflammatory and membrane stabilizing activities of the isolated compound.

## Experimental

*Plant materials*

The Plant specimen for the proposed study was collected from Melmaruvattur Chennai, Tamil Nadu. It was identified and authenticated by Dr. P. Jayaraman, Director, Plant Anatomy Research Center, (PARC) Tambaram, Chennai. A voucher specimen (accession No. 31238) was deposited in the Herbarium for future reference.

*Extraction and isolation of alkaloid*

The coarsely powdered root bark of *Plumeria acutifolia *(2 Kg) was exhaustively extracted with methanol (5 × 1 L) at room temperature. The methanol extract (40 g) was acidified (pH = 2) with 2 M hydrochloric acid and the final volume was adjusted to 400 mL. The aqueous acidic solution was then extracted with ethyl acetate (3 × 200 mL) to remove neutral components. After the removal of neutral components, the aqueous layer made alkaline (pH = 9) with 30% ammonium hydroxide solution and was repeatedly extracted with chloroform (3 × 300 mL). The combined extracts were evaporated under vacuum to yield the crude alkaloid (5.4 g). The crude alkaloid was chromatographed over the silica gel (60-120 mesh size) and eluted with solvents of increasing polarity viz., hexane, chloroform and methanol. A total of 150 fractions, 25 mL each, were sampled and their homogeneity determined by TLC, using a solvent system of Toluene : chloroform : ethanol (4 : 4 : 12).

Compound 1 (320 mg) was isolated from chloroform (60%) and methanol (40%) fraction as yellow amorphous powder which resulted in a positive test with the dragendorff reagent, R_f_ 0.874. The compound 1 was characterized and identified by analyzing its spectral data. The ^1^H NMR spectrum showed the presence of protons between 7.4 and 8.6 ppm, due to the presence of an aromatic moiety or double bonds. Two protons with a chemical shift higher than 8 ppm suggested a pyridine ring, unsubstituted in both *ortho *positions of the nitrogen atom. This was in agreement with the ^13^C NMR spectra, where two CH signals at 150.85 and 145.65 ppm were present. In addition, the typical ^13^C signals for an *α, β*-unsaturated spirolactone moiety were observed. Other structural features, evident from 1H and ^13^C NMR spectra, were a –CH_2_-CH_2_- moiety and a CH_3_-CH-OH moiety. Complete assignments based on the two-dimensional NMR spectra are listed in Table 1. The Electrospray ionization (ESI) mass spectrum recorded in positive ion mode showed an [M + H]^+ ^peak at *m/z *= 232 and an [M + Na]^+^ peak at *m/z *= 254, which were in agreement with the expected molecular weight of 231 (C_13_H_13_NO_3_).

**Table 1:** ^1^H (400 MHz) and ^13^C NMR (100 MHz) assignments of Plumerianine

**Carbon No**	^13^ **C NMR [*δ *(ppm)]**		^1^ **H NMR [** ***δ *** **(ppm), mult., J (Hz)]**	
	**Major**	**Minor**		
	145.65	145.53	8.22, s	8.27, s
**3**	150.85	150.89	8.49, d, 5.0	8.49, d, 5.0
**4**	122.44	122.44	7.47, m*	7.47, m*
**5**	157.03	157.03	-	-
**6**	31.09	31.12	3.25, m/3.15, m/	3.25, m/3.15, m/
**7**	36.76	36.76	2.62, m/2.42, m	2.62, m/2.42, m
**8**	94.90	94.85	-	-
**9**	137.84	137.91	-	-
**10**	150.15	150.15	7.47, m*	7.47, m*
**11**	139.88	139.90	-	-
**12**	172.85	172.91	-	-
**13**	63.73	63.61	4.65, m	4.65, m
**14**	22.44	22.26	1.49, d, 6.6	1.45, d, 6.6

* Overlapping signals

*Animals*

Wistar rats (150-200 g) and Swiss albino mice (18-25 g) of either sex obtained from the Laboratory Animals Center, Vels University, were used for the various studies. They were kept in a well-ventilated laboratory environment (a light-dark cycle (12 h-12 h)), for seven days for acclimatization and had free access to food and water *ad libitum*. Animals were fasted overnight and weighed before the experiment. 

*Acute toxicity study*

Acute toxicity study (up-and-down procedure) was carried out as per the guidelines by Organization for Economic Co-operation and Development (OECD) 423 (9). Mice (6 per group) were divided into six groups. The first 5 groups received oral doses of 100, 200, 300, 400 and 500 mL/Kg of isolated compound 1. The sixth group received saline (10 mL/Kg) orally. Mortality was assessed 24 h after the administration. The animals were also observed for toxic symptoms and mortality was determined 24 h after the treatment.

*Anti-allergic activity*

*Study on passive cutaneous anaphylaxis*

*Preparation of antiserum from rats*

The Wistar rats of either sex were injected intra-peritoneally with 0.2 mL, 10% egg albumin, 0.2 mL of bordetella pertussis vaccine on the 1^st^, 3^rd^, and 5^th^ day. After 21 days of the first immunization, blood was collected from orbital plexus under light ether anesthesia. The blood was allowed to clot and serum was separated by centrifugation at 1500 rpm. The separated serum was stored at 20°C until it was used for the experiment (10).

Rats (6 per group) were divided into five groups. The first 3 groups received oral doses of 10, 25 and 50 mL/Kg of the compound 1. The 4^th^ and 5^th^ groups were treated orally with ketotifen (5 mL/Kg p.o) as a reference drug and saline (10 mL/Kg) as control respectively. 

The anti ovalbumin serum was injected intra-dermally on the dorsal skin of the animal. The drug/extract was administered to animal according to their group for three consecutive days from the day of sensitization. After the treatment, 1 mL of 0.5% Evans Blue solution containing 20 mg of egg albumin was injected intra-venously through the tail vein. Because of antigen-antibody reaction, there was increased vascular permeability and dye will penetrate in that tissue area. This area of skin was removed after being sacrificed. The skin portion was transferred to the solution of 70% acetone for 24 h. The dye was extract out in the acetone and Evans Blue dye was measured colorimetrically at 620 nm. The amount of dye penetrate in the skin area reflects the severity of hypersensitivity reaction. The inhibition% was calculated through the following formula: 

(C – T / C) × 100 

*Study on passive paw anaphylaxis*

*Preparation of antiserum from rats*

The Wistar rats of either sex were injected intra-peritoneally with 0.2 mL, 10% egg albumin, 0.2 mL of bordetella pertussis vaccine on the 1^st^, 3^rd^, and 5^th^ day. After 21 days of the first immunization, blood was collected from the orbital plexus under the light ether anesthesia. The blood was allowed to clot and serum was separated by centrifugation at 1500 rpm. The separated serum was stored at 20°C until it was used for the experiment (11).

*Passive paw anaphylaxis*

Rats (6 per group) were divided into five groups. The first 3 groups received oral doses of 10, 25 and 50 mL/Kg of the compound 1. The 4^th^ and 5^th^ groups were treated orally with indomethacin (10 mL/Kg) as a reference drug and saline (10 mL/Kg) as control respectively. The animals were dosed for seven consecutive days. 

Two hours after the last dose of drug administration (on seventh day), rats were passively sensitized into the left hind paw with 0.1 mL of the undiluted serum. The contralateral paw received an equal volume of saline. Twenty-four hours after the sensitization, the rats were challenged in the left hind paw with 10 mg of egg albumin in 0.1 mL of saline. The hind paw volume was measured after 30 min by volume displacement method using mercury column plethysmometer. The inhibition% was calculated using the previously mentioned formula. 

**Figure 2:**
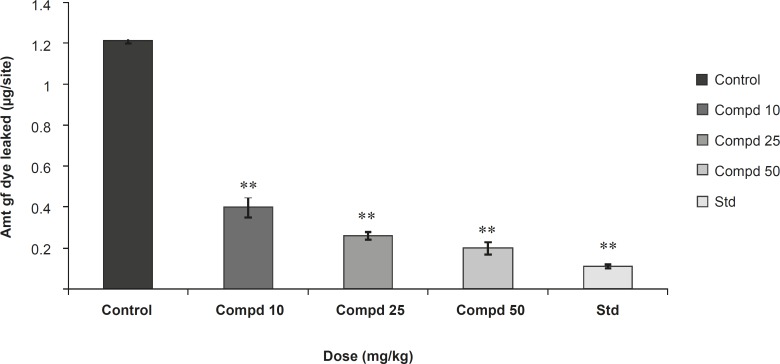
Study of compound 1 on passive cutaneous anaphylaxis (PCA).

**Figure 3:**
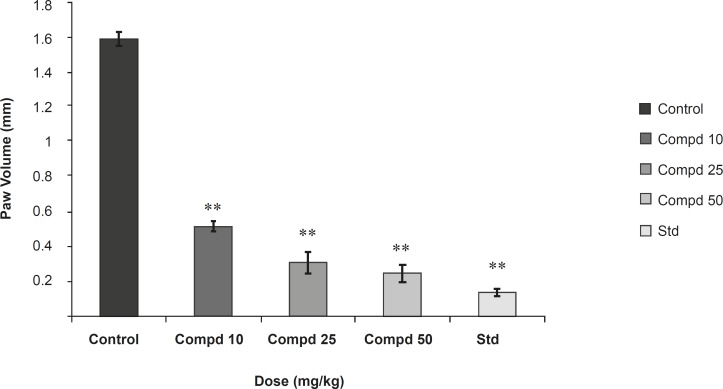
Study of compound 1 on passive paw anaphylaxis (PPA). Values are mean ± SEM of 6 parallel measurements. Statistical significant test for comparison was done by ANOVA, followed by Dunnett’s ‘t’ test (n = 6). All the values are significant. **: p < 0.01 when compared against control.

Results were presented in Figures 2 and 3. 

*Anti-inflammatory activity*

*Acute model*

**Figure 4:**
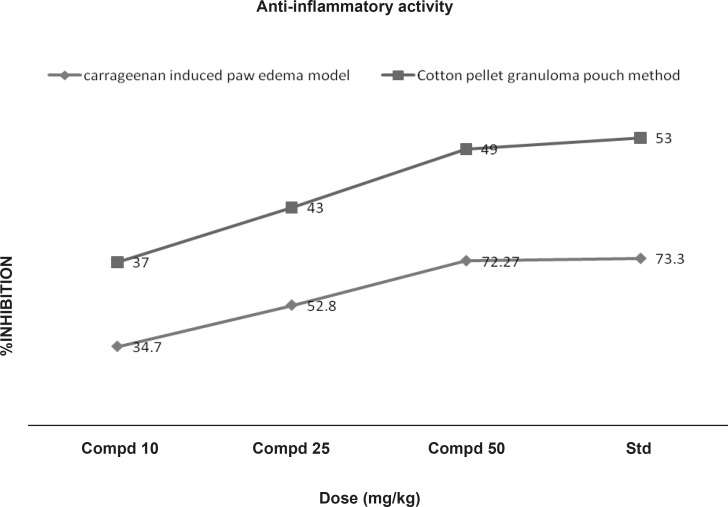
Effects of compound 1 on Carrageenan induced paw edema and cotton pellet granuloma pouch method

*Carrageenan induced paw edema* ***: ***The anti-inflammatory activity was measured by using carrageenan-induced rat paw edema model. Rats (6 per group) were divided into five groups. The first 3 groups received oral doses of 10, 25 and 50 mL/Kg of the compound 1. The 4^th^ and 5^th^ groups were treated orally with indomethacin (10 mL/Kg) as a reference drug and saline (10 mL/Kg) as the control respectively. Acute inflammation was produced by subplantar injection of 0.1 mL of 1% suspension of carrageenan in normal saline, in the right hind paw of the rats, 1 h after the oral administration of the test compound (10, 25 and 50 mL/Kg. p.o.). Indomethacin at a dose of 10 mL/Kg, p.o. was used as standard anti-inflammatory drug. The paw volume was measured plethysmometrically (at 1, 2, and 3 h) after the carrageenan injection (12). Results were expressed as the percentage of inhibition of edema, shown in Figure 4, calculated by the following formula: 

(1 − V_t_ /V_c_) × 100 

In this formula, Vt and Vc are the mean paw volume in the treated and controlled groups, respectively. 

*Chronic model*

*Cotton pellet granuloma pouch method*

Chronic inflammation was induced by cotton pellet granuloma. Rats (6 per group) were divided into five groups. The first 3 groups received oral doses of 10, 25 and 50 mg/Kg of the compound 1. The 4^th ^and 5^th ^groups were treated orally with indomethacin (10 mL/Kg) as a reference drug and saline (10 mL/Kg) as control, respectively. Autoclaved cotton pellet 50 ± 1 mg was implanted subcutaneously by making incision in the axilla and groin region of each rat under the ether anesthesia. Drugs were administered orally for 7 consecutive days from the day of cotton pellet implantation. Animals were sacrificed on 8^th^ day and the granuloma was dissected out, dried in an oven at 60°C for 24 h and weighed. The increment in the dry weight of the pellet was taken as a measure of granuloma formation (13) . The percentage of inhibition of the granuloma was shown in Figure 4, determined using the following formula: 

(1 − W_t_ / W_c_) × 100 

Where: W_t_ = Dry weight of the cotton in test animals and W_c_ = Dry weight of the cotton in control animals. 

*Membrane stabilizing activity*

*Preparation of erythrocyte suspension*

Whole blood was obtained with heparinized syringes from rats through cardiac puncture. The blood was washed three times with isotonic buffered solution (154 mM of NaCl) in 10 mM sodium phosphate buffer (pH = 7.4). The blood was centrifuged each time for 10 min at 3000 g (14). 

*Hypotonic solution-induced rat erythrocyte hemolysis*

**Figure 5:**
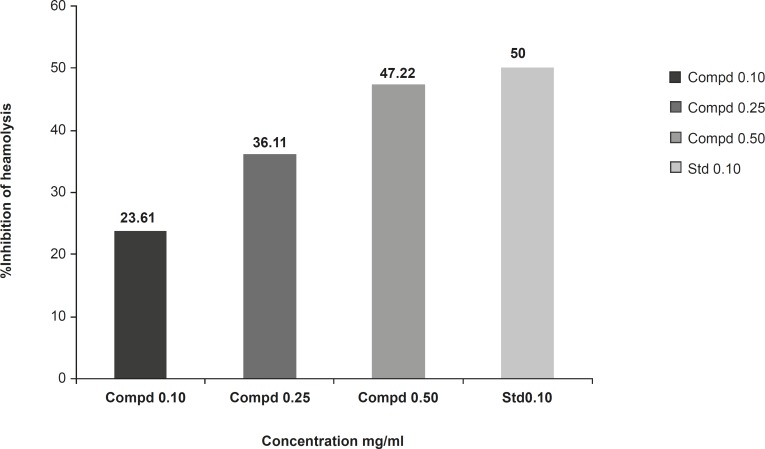
Effect of the compound 1 on rat erythrocyte hemolysis induced by hypotonic solution.

Membrane stabilizing activity of the extract was assessed using hypotonic solution-induced rat erythrocyte hemolysis. The test sample consisted of stock erythrocyte (RBC) suspension (0.50 mL) mixed with 5 mL of hypotonic solution (50 mM of NaCl) in 10 mM of sodium phosphate buffered saline (pH = 7.4) containing the compound 1 (10, 25 and 50 mg/mL) or indomethacin (0.1 mg/mL). The control sample consisted of 0.5 mL of RBC mixed with hypotonic-buffered saline solution alone. The mixtures were incubated for 10 min at room temperature and centrifuged for 10 min at 3000 g and the absorbance of the supernatant was measured at 540 nm. The percentage inhibition of hemolysis or membrane stabilization presented in Figure 5, calculated by the following formula: 

100 × (OD1-OD2/OD1) 

Where: OD_1_ = Optical density of hypotonic-buffered saline solution alone; OD_2_ = Optical density of test sample in hypotonic solution*. *

*Statistical analysis*

The results were analyzed for statistical significance using one-way analysis of variance (ANOVA) followed by Dunnett’s test. Values with p < 0.05 were considered significant. 

## Results and Discussion

Compound 1 was isolated as amorphous yellow powder and showed a molecular ion peak at *m/z *= 232 ([M + H]^+^) and 254 ([M + Na]^+^), which were in agreement with the expected molecular weight of 231 (C_13_H_13_NO_3_). ^1^H-NMR and 13C-NMR spectra of compound showed characteristics of a lupine alkaloid plumerianine as shown in Figure 1. 

*Plumerianine*

(R)-4’-[(S)-1 hydroxy ethyl)-5, 6-dihydro- 5’H-spiro [cyclopenta [c]pyridine-7, 2’furan]- 5’-one}, [α]^25^_D_: +18 (c 0.18, CHCl_3_); ^1^H-NMR and 13C –NMR: given in Table 1; ESI-MS: *m/z *= 254 ([M + Na]^+^), 232 (([M + H]^+^). 

*Acute toxicity studies*

Acute toxicity study showed that the compound 1 possessed high safety profile as no death was observed at oral doses of 100-500 mL/Kg in mice. 

*Anti-anaphylactic activity*

In the study on passive cutaneous anaphylaxis model**, **compound 1 produced a significant dose dependent decrease in the amount of Evans Blue dye leaked at site in comparison with the control. Standard drug also makes a significant decrease in the amount of Evans Blue dye leaked at site and in passive paw anaphylaxis model. Compound 1 produced a significant dose-dependent decrease in the paw volume induced by antiserum (Figures 2 and 3).

*Anti-inflammatory activity*

In acute model of inflammation (carrageenan induced), paw edema volume is dose-dependently inhibited by the compound 1 (10, 25 and 50 mL/ Kg p.o). As shown in table 1, the compound 1 (50 mL/Kg) reduced the oedema swellings by 72.27% as compared with 73.33% reduction produced by indomethacin (10 mL/Kg, p.o) at 3^rd^ h of the carrageenan administration. In chronic model of inflammation, compound 1 at doses of 10, 25 and 50 mL/Kg exhibited significant (p < 0.01) reduction in granuloma weight by 37, 43 and 49% respectively. These results were comparable with that of the standard drug (Figure 4). 

*Effect on erythrocyte membrane stability*

The compound 1 (at concentration range of 0.10-0.50 mg/mL) significantly protected the rat erythrocyte membrane against lysis induced by hypotonic solution. At a concentration of 0.50 mg/mL, the extract produced 47.22% inhibition of RBC hemolysis as compared with 50% produced by indomethacin **(**Figure 5). 

The present study was undertaken for the evaluation of anti-anaphylactic activity and anti-inflammatory property of a compound isolated from the root bark of *Plumeria acutifolia*. The anti-anaphylactic activity was done using passive cutaneous anaphylaxis for evaluation of compound plumerianine on immediate hypersensitivity reaction. Mediators like leukotriene, prostaglandins, PAF and cytokines are reported to be responsible for the immediate hypersensitivity reaction, but enhanced vascular permeability and leukocyte infiltration at the sites of allergen challenge were observed. In our model, anti-ovalbumin serum obtained from sensitized rats was injected to the rats. The enhanced vascular permeability was estimated by Evans Blue dye. The leakage of dye was significantly less in the rats treated with compound plumerianine (10, 25 and 50 mL/Kg) than the control animals. This activity can be partly due to the inhibition of leukotriene synthesis. Passive paw anaphylaxis is another *in-vivo *model for IgE-mediated immediate hypersensitivity reactions (15). In both these models, a prominent inhibitory effect of plumerianine is suggestive of its antianaphylactic activity (Figures 2 and 3).

The results of the study showed that the compound plumerianine possesses anti-inflammatory property, as it significantly inhibited oedema induced by carrageenin, granuloma tissue formation in rats and offer significant protection of the erythrocyte against the lysis induced by hypotonic solution.

The inflammatory condition induced by carrageenin involves step-wise release of vasoactive substances such as histamine, bradykinin and serotonin in the early phase and prostaglandins in the acute late phase. These chemical substances produced an increase in vascular permeability and thereby, promoting accumulation of fluid in tissues that accounts for the oedema (16). The cotton pellet method is widely used to evaluate the transudative and proliferative components of the chronic inflammation. The dry weight of the pellets correlates with the amount of the granulomatous tissue (17, 18). Administration of compound 1 (10, 25 and 50 mL/Kg b.w) appeared to be effective in inhibiting the dry weight of the cotton pellet that was almost near to that of indomethacin (Figure 4). These data support the hypothesis of the greater effect of the compound 1 on the inflammation in rats. This effect may be due to the cellular migration to injured sites and accumulation of collagen and mucopolysaccharides. 

The compounds with the membrane-stabilizing properties are well known for their ability to interfere with the early phase of inflammatory reactions, namely the prevention of phospholipases release that triggers the formation of inflammatory mediators (19). The compound 1 demonstrate significant membrane stabilizing property (Figure 5), which suggests that its anti-inflammatory activity observed in this study, may be related to the inhibition of the late phase of inflammatory events, namely the release of chemical mediators.

Thus, the results of this study demonstrated that the alkaloid plumerianine has significant anti-anaphylactic and anti-inflammatory activities. However, a more extensive study is necessary to determine the exact mechanism (s) of the action.
